# Supplementary material for: Recommendations for developing asynchronous online consultations for chlamydia treatment for underserved populations: a Behaviour Change Wheel analysis
Source: Sex Transm Infect. 2026 Jan 8;102(6):e056677. doi: 10.1136/sextrans-2025-056677 (PMC13539832; doi:10.1136/sextrans-2025-056677)
Supplement: Supplementary file 3 [file sextrans-102-6-s002.docx]

**Supplementary File 2. Information Power criteria**

| **Information Power items and dimensions** | | **Our study** |
| --- | --- | --- |
| Study aim – narrow or broad? | Information power, guiding adequate sample size is related to the study aim. A broad study aim requires a larger sample than a narrow aim to offer sufficient information power, because the phenomenon under study is more comprehensive. | Narrow – the aim is highly specific. |
| Sample specificity – dense or sparse? | To offer sufficient information power, a less extensive sample is needed with participants holding characteristics that are highly specific for the study aim compared with a sample containing participants of sparse specificity. Specificity concerns here participants who belong to the specified target group while also exhibiting some variation within the experiences to be explored. | Dense – characteristics of the sample meet those of the target population and study aim (people underserved by current sexual health provision) (Table 1) |
| Established theory – applied or not? | Information power, guiding adequate sample size is related to the level of theoretical background of the study. A study supported by limited theoretical perspectives would usually require a larger sample to offer sufficient information power than a study that applies specific theories for planning and analysis. | Theory applied – PROGRESS+ used for purposive sampling; BCW used to develop interview schedule and analysis. Theories allowed for targeted collection of people from underserved populations and development of highly specific interview questions to address study aim. |
| Quality of dialogue – strong or weak | Information power is also related to the quality of the interview dialogue. A study with strong and clear communication between researcher and participants requires fewer participants to offer sufficient information power than a study with ambiguous or unfocused dialogues. | Strong – Data were collected by an experienced qualitative researcher (JM) under the supervision of a qualitative research expert (PF) and sexual health clinician and research expert (CSE) with daily (JM and PF) or weekly (JM, PF, and CSE) check ins about the specify of data being collected. Collected data indicate that participants had highly relevant specific and varied experiences related to the study aim (Tables 2 and 3). |
| Analysis strategy – case or cross-case? | Information power is related to the strategy chosen for analysis in the specific project. An exploratory cross-case analysis requires more participants to offer sufficient information power compared with a project heading for in-depth analysis of narratives or discourse details from a few, selected participants. | Cross-case – The thematic analysis, conducted by an experienced qualitative researcher under the supervision of a qualitative research expert and reviewed by sexual health specialists, identified barrier and facilitator themes across participants from underserved populations and captured both depth and variation in experiences relevant to the study aim (Tables 2-3). |
