# Supplementary material for: Recommendations for developing asynchronous online consultations for chlamydia treatment for underserved populations: a Behaviour Change Wheel analysis
Source: Sex Transm Infect. 2026 Jan 8;102(6):e056677. doi: 10.1136/sextrans-2025-056677 (PMC13539832; doi:10.1136/sextrans-2025-056677)
Supplement: Supplementary file 4 [file sextrans-102-6-s003.docx]

**Eligibility Questions**

1. Are you aged 16 or over?

2. Do you live in the UK?

3. Do you have you have access to the internet?

4. Have you had sex in the last 12 months?

5. a) Have you ever ordered a postal STI self-sampling kit before?

b) *[If yes]* Did you experience any difficulty in ordering it?

**PROGRESS+ screening questions:**

1. What age are you?

2. How would you best describe your gender?

3. Were you assigned a different gender at birth?

4. How would you describe your sexual orientation?

5. How would you describe your ethnicity?

6. What is your highest educational qualification?

7. How would you describe your current occupation?

8. What is your postcode?

9. a) Do you have any physical or mental health conditions or illnesses that have lasted or are expected to last for 12 months or more?

b) *[If yes]* Do any of your illnesses or conditions reduce your ability to carry out day to day activities?

10. Do you consider yourself to have a learning disability?

**PROGRESS+ demographic questions:**

1. a) Were you born in the UK?

b) *[If no]* Which country were you born in?

2. What is your first language?

3. a) Do you regard yourself as belonging to any particular religion or faith?

b) *[If yes]* Which religion or faith?

4. Do you ever have difficulty making ends meet at the end of the month?

5. How many people can you call upon to provide support if you need it?

**Internet access and use questions:**

1. How do you typically access the Internet?

2. What device(s) do you use to access the internet?

3. Are these your own devices?

4. How often do you go online?

5. What sorts of things do you go online for?

6. a) Have you ever searched for ~~health or~~ sexual health information online?

b) *[if yes]* What sort of information have you searched for?

7. a) Have you ever used any online ~~health or~~ sexual health services?

b) *[if yes]* Which services have you used?

- prompt about live chat/email or text service

8. How would you rate your skills in using the internet?
